# Recent Advances in Hydrogel-Based Sensors Responding to Ionizing Radiation

**DOI:** 10.3390/gels8040238

**Published:** 2022-04-12

**Authors:** Ping Zhang, Li Jiang, Hong Chen, Liang Hu

**Affiliations:** 1 State and Local Joint Engineering Laboratory for Novel Functional Polymeric Materials, College of Chemistry, Chemical Engineering and Materials Science, Soochow University, Suzhou 215123, China; 20204209028@stu.suda.edu.cn (P.Z.); chenh@suda.edu.cn (H.C.); 2 State Key Laboratory of Radiation Medicine and Protection, School for Radiological and Interdisciplinary Sciences (RAD-X) and Collaborative Innovation Center of Radiation Medicine of Jiangsu Higher Education Institutions, Soochow University, Suzhou 215123, China; 20194220028@stu.suda.edu.cn

**Keywords:** gel, Fricke, dosimeter, radio-chromic, radio-fluorescent, nanoparticle, polyacrylamide

## Abstract

Ionizing radiation and its applications are widely spread throughout life. Similar to many other things, both the positive and negative aspects of ionizing radiation should always be kept in mind. For example, a proper radiation dose can be delivered to tumor tissue to kill malignant cells in radiotherapy. On the other hand, exceeding this dose can damage the normal tissues of a human organism. Therefore, the application of sensors for measuring ionizing radiation doses is of utmost importance in many fields, especially in cancer therapy. Traditional dosimeters, such as ionization chambers, silicon diodes and thermoluminescence dosimeters, are widely used. However, they have limitations in certain aspects. Hydrogel-based sensors (or dosimeters) for measuring ionizing radiation doses attract extensive attention for decades due to their equivalence to living tissue and biocompatibility. In this review, we catalog hydrogel-based dosimeters such as polymer, Fricke, radio-chromic, radio-fluorescence and NPs-embedded dosimeters. Most of them demonstrate desirable linear response and sensitivity regardless of energy and dose rate of ionizing radiation. We aim to review these dosimeters and their potential applications in radiotherapy as well as to stimulate a joint work of the experts from different fields such as materials science, chemistry, cancer therapy, radiobiology and nuclear science.

## 1. Introduction

Radiation can be classified as non-ionizing or ionizing. Ionizing radiation can activate or ionize atoms in the matter with which they interact. Ionizing radiation is widely used in industry, agriculture, medicine and national defense [1,2,3]. The applications in radiotherapy imply delivery of the adequate dose of ionizing radiation (i.e., photons, electrons or neutrons) to the tumor tissue to kill malignant cells, and at the same time minimizes the dose for the surrounding normal tissues. Therefore, measuring the dose of ionizing radiation before and/or during the radiotherapy treatments is an effective method to ensure the quality of treatment and the radiation protection of a patient.

**Figure 1:**
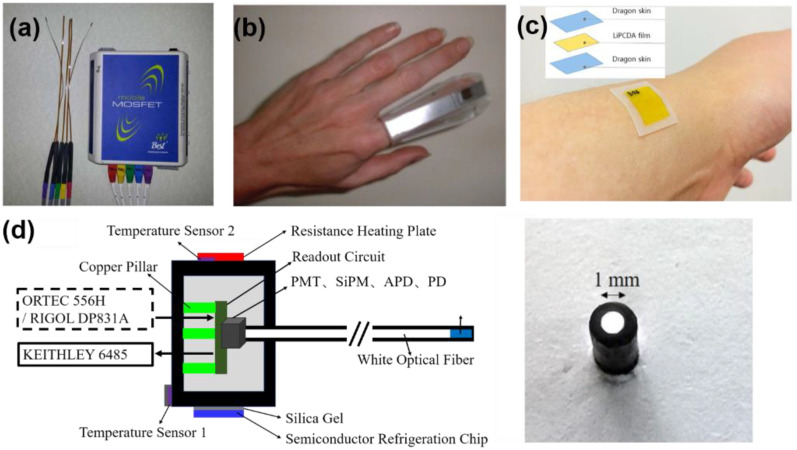
(**a**) MOSFETs from ref. [4] with permission from John Wiley and Sons, (**b**) a finger TLD, adapted from ref. [5] with permission from Elsevier, (**c**) flexible film dosimeter, adapted from ref. [6] with permission from John Wiley and Sons and (**d**) schematic of a fiber dosimeter and a photograph of the polished end-face of a white optical fiber from ref. [7] with permission from Springer.

The sensors that are applied to accurately measure radiation doses are often called dosimeters. They include metal-oxide-semiconductor field-effect transistors (MOSFETs), silicon diodes, films, ionization chambers and thermoluminescence dosimeters (TLDs), as well as many others (Figure 1) [4,5,6,7,8,9,10]. MOSFETs are expensive and operate at high voltages, which makes them unsuitable for in situ dose measurements. The advantages of silicon diodes are their simple structure, excellent sensitivity and high spatial resolution. However, the results of the dose measurements by them depend on irradiation angle, beam energy and dose rate. Moreover, such diodes have poor tissue equivalence, temperature and time-instability, as well as a large batch–batch difference. TLDs have poor mechanical strength, and their usage involves complex annealing processes. In addition, the above-mentioned dosimeters often require sophisticated preparation technologies and operation conditions, which restrict their applications.

**Figure 2:**
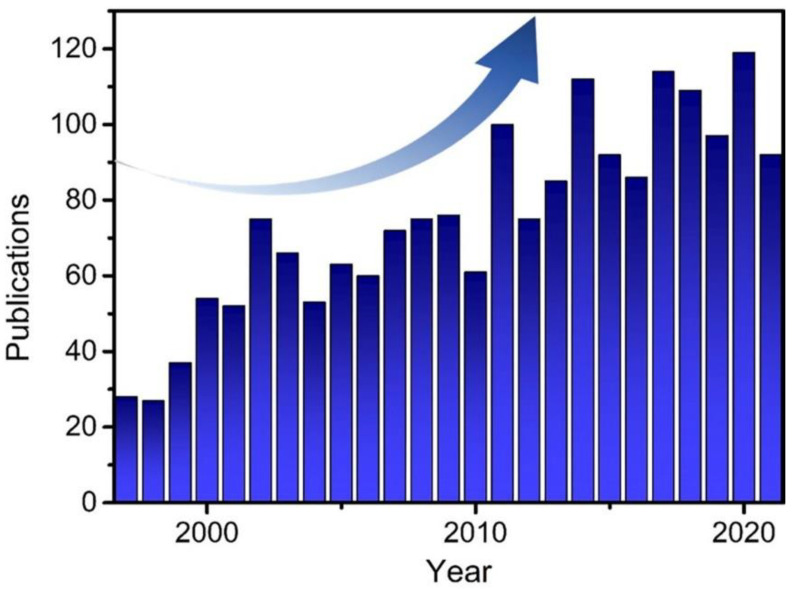
The number of publications regarding gel dosimeters from 1997 to February 2022, which was searched in Web of Science using Gel AND dosimet* as the topic.

The polymer hydrogel has a unique three-dimensional network structure, containing a large amount of water. Owing to their excellent tissue equivalence and biocompatibility, hydrogel (or gel) dosimeters attract extensive attention [11,12,13,14,15]. Since the 1950s, various such dosimeters were developed, including Fricke, polyacrylamide (PAAm) and radiochromic gel dosimeters. Figure 2 presents the numbers of publications devoted to gel dosimeters in the period from 1997 to February 2022 found in the Web of Science by Gel AND dosimet* keywords, which clearly show an increasing trend. Recently, several good reviews devoted to gel dosimeters were published [11,12,13,14,15]. For instance, Marrale et al. made a review of both Fricke and PAAm gels, focusing on their chemical and physical properties and applications for radiation dosimetry [11]. Nezhad et al. systematically reviewed low-energy radiation applications of gel dosimeters [14]. Farhood et al. reviewed gel dosimeters as the devices for different radiotherapy applications [12]. In this work, we summarize the information on polymer hydrogel and Fricke hydrogel dosimeters. Moreover, we review radio-chromic and radio-fluorescent hydrogel dosimeters as well as NPs-embedded ones, mostly using the literature sources published in the last 5 years. We expect more experts from different fields of science such as materials science, chemistry, cancer therapy, radiobiology and nuclear science to work together to achieve clinical applications of gel dosimeters.

## 2. Gel Dosimeters

### 2.1. Polymer Dosimeters

Polymer hydrogel dosimeters are usually prepared by dissolving reactive monomers containing carbon–carbon double bonds in a gel matrix [16]. The monomers are initiated and polymerized by ionizing radiation, which results in the changes in their physical and chemical properties, such as turbidity, density and elasticity. These changes are closely related to the dose absorbed by the dosimeter. Therefore, the absorbed dose value can be obtained by magnetic resonance imaging (MRI), X-ray computed tomography (CT), optical-CT (OCT), ultrasound and optical scanning (Table 1) [17,18,19,20,21].

Polyacrylamide gel (PAG) dosimeters belong to the most common ones. They are also known as BANANA gel dosimeters. Their composition includes AAm (reactive monomer), nitrous oxide (N_2_O), agarose and BIS (as crosslinkers) and water [22]. The addition of N_2_O gas prior to use enables one to eliminate oxygen gas from the solution and to avoid oxygen-induced retardation of polymerization after irradiation. Under 6 MV X-ray irradiation, the polymerization in agarose occurred, which results in the change of the proton relaxation time of water molecules. The value of relaxation rate R1 linearly increases with the increase in X-ray dose from 0 to 20 Gy. Such dosimeters show high spatial resolution and long-term stability of dose signals. 

**Figure 3:**
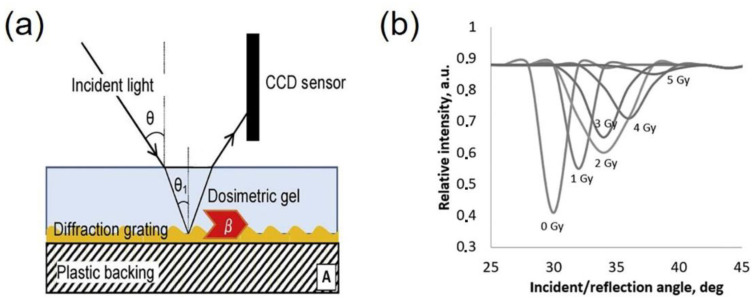
(**a**) Schematic illustration and (**b**) responsivity of SPR-based gel dosimeter, adapted from ref. [25] with permission from Elsevier. In (**a**), θ is resonant incident angle, β is the plasmon propagation constant, CCD is charge coupled device, respectively.

As compared to BANANA gels, the BANG ones that are composed of AAm, BIS, gelatin and water, are more transparent, therefore being advantageous for optical dosimetry [23,36]. Irradiation-induced polymerization of BANG gels affects the mobility of surrounding water molecules and, hence, the spin–spin relaxation (R2) rate of NMR. Polymer dosimeters are suitable for quality assurance in radiotherapy [37,38,39]. Recently, Kudrevicius et al. used modified PAG dosimeters for quality assurance in stereotactic radiosurgery treatments. Compared to radio-chromic film dosimetry, PAG dosimeters demonstrated excellent sensitivity, spatial resolution and linear dose response in the hypofractionated (>10 Gy) dose range [38]. Adliene et al. reported a surface plasmon resonance (SPR) sensor for measuring radiation doses (Figure 3) [25]. The authors deposited nPAG gel (its composition included AAm, BIS, gelatin and tetrahydroxymethyl chloride) as an antioxidant with the thickness ~5 μm onto the surface of Au gate, followed by polymerization induced by the X-ray irradiation. As the X-ray dose increased from 0 to 5 Gy, the SPR peak position gradually shifted to higher values during the next 24-h post-irradiation period. The limit of detection (LOD) of such a dosimeter was as low, at 0.2 Gy, and its sensitivity was 0.168 Gy^−1^.

As oxygen gas can inhibit the polymerization process, PAG gel dosimeters should be prepared and used in an oxygen-free environment. These dosimeters usually are stored in glassware or plastic vessels due to their low oxygen permeability [40]. Moreoever, adding antioxidants in the polymer dosimeters can be effective in scavenging the oxygen, such as AA, N-acetyl-cysteine and tetrakis (hydroxymethyl) phosphonium chloride [41]. Furthermore, such dosimeters are toxic due to the toxicity of AAm moiety in their composition. In this regard, many other monomers with less toxicity, such as NIPAm, AAc, MAA and DEMA, were used (Table 1). Fong et al. reported a MAGIC gel dosimeter that consists of MAA, gelatin, CuSO_4_, AA, hydroquinone and water [42]. After γ-ray irradiation, an AA-Cu^2+^ complex consumes oxygen from the system and forms free radicals to initiate the polymerization of MAA. Therefore, such dosimeters can be prepared in an aerobic environment. The Addition of a small amount of hydroquinone facilitates their storage and prevents the polymerization of MAA before exploitation. The results of the investigations of such dosimeters demonstrate that the R2 signal in MRI increases linearly in the range of the irradiation doses of 0–30 Gy, and the sensitivity reaches 0.681 s^−1^ Gy^−1^.

Later, various other polymer gel dosimeters, such as NIBMAGAT [34] and NHMA [35] ones, were proposed and studied. Rabaeh et al. reported a N-(isobutoxymethyl) acrylamide gel dosimeter (NIBMAGAT) [34]. The R2 sensitivity of NIBMAGAT reached 0.4776 s^−1^ Gy^−1^ in the X-ray dose range of 0–10 Gy, due to the higher NIBMA concentration in the water–acetone cosolvent. The measurement capability of this dosimeter was independent of the energy and dose rate and it had a long storage time (~10 days). Jaszczak et al. reported a polymer gel dosimeter containing N-vinylpyrrolidone and Pluronic F-127 [poly(ethylene oxide)-block-poly(propylene glycol)-block-poly(ethylene oxide), PEO-PPO-PEO, approved by the Food and Drug Administration, US] [43]. Pluronic F-127 ensured the improved transparency of the gel, making it possible to use the optical X-ray CT to read the post-irradiation dosimeter information. The linear dose response of the dosimeter being considered was in the range of 1–20 Gy, the dynamic dose range was 1–50 Gy and the threshold dose was ~1 Gy, respectively. Moreover, its dose response was independent of the radiation dose rate, energy and type (photons and electrons). Furthermore, the dosimeter was stable for at least 8 days after irradiation due to the Pluronic F-127, which is highly important for its storage. 

To improve the sensitivity of gel dosimeters, nanoparticles (NPs) of elements with high atomic numbers [30,44,45,46], inorganic salts [35,47,48] and urea [26] are used as their components. Mustaqim et al. reported a MAGAT gel dosimeter doped by ZnO NPs and methylene blue [30]. The latter substance can act as an inhibitor in the absence of oxygen, which reduces the self-polymerization ability of the MAGAT gel. Besides, ZnO NPs, owning to the high atomic numbers of their elements, increase the cross-section of photoelectric effect and, thus, enhance the dose absorbed by the dosimeter. The dose enhancement factor was found to be 1.03 in the presence of ZnO NPs, reflecting higher dosimeter sensitivity. Furthermore, Rajaee et al. introduced BiFeO_3_ NPs into the MAGIC gel dosimeter [28]. Monte Carlo simulations demonstrated that these NPs had a significant radiation-enhanced sensitization effect in low-dose rate brachytherapy (^125^I), again due to the high atomic numbers of the elements composing them. It is worth noticing that BiFeO_3_ NPs have a magnetic thermal effect; thus, a controllable drug release from the gel by radiation can be achieved, thus enabling one to combine radiotherapy and chemotherapy.

It was also reported that the addition of a small amount of LiCl was enabled to improve the sensitivity of the N-(Hydroxymethyl)acrylamide gel dosimeters by 50% [35]. The underlying mechanism consists in the binding of metal ions with water and monomer molecules by electrostatic interaction, which yields more free radicals under irradiation. This effect increases the polymerization reaction rate, thereby improving the dosimeter sensitivity. Moreover, urea was introduced to the U-PNIPAm gel dosimeter (composed of BIS, NIPAm, gelatin, TPHC and water) to enhance its sensitivity, which depended on the photon energy (6 and 15 MV), but was independent on the dose rate. The dosimetric resolution of the U-PNIPAM gel dosimeter achieved the values of 0.06–0.20 Gy [26].

### 2.2. Fricke Gel Dosimeters

Fricke gel dosimeters are based on the conversion of ferrous (Fe^2+^) ions to ferric (Fe^3+^) ones by ionizing radiation. The concentration of Fe^3+^ is linearly proportional to the absorbed radiation dose. It can be measured using optical spectroscopy or NMR. Fricke gel dosimeters have many advantages, such as easy fabrication, safety, reliability and low cost. Gelatin was first used as a gel agent to prepare such dosimeters. Fe^2+^ and Fe^3+^ are paramagnetic contrast agents with different electron spin relaxation times, which have different effects on NMR relaxation rate. Therefore, NMR measurements of the relaxation times of dosimeters can be used to determine the radiation doses by calculating spin relaxation times T1 values before and after irradiation [49]. However, the performance of Fricke gelatin dosimeters is deteriorated by Fe^2+^ ion autoxidation and the diffusion of Fe^3+^ ions [50,51]. Furthermore, the gelatin matrix undergoes phase transformation at ~30 °C, which limits the storage and transportation of the dosimeters of this kind.

PEO and poly(vinyl alcohol) (PVA) are commonly used as alternative matrices to prepare Fricke dosimeters to overcome the problem of the diffusion of Fe^3+^ ions. PEO and PVA are inexpensive, clean and suitable for large-scale industrial production [50,52,53]. It was found by optical and MR studies that the PVA-Fricke gel dosimeters can detect γ-rays doses in the range of 0–20 Gy with the sensitivity of 0.046 Gy^−1^ and 0.02 s^−1^ Gy^−1^, respectively [53]. Araujo et al. reported a PEO-Fricke gel dosimeter consisting of PEO, Fe(NH_4_)_2_(SO_4_)_2_∙6(H_2_O), H_2_SO_4_ and NaCl. PEO increased the density of the gel matrix and reduced the diffusion of Fe^3+^ ions [50]. When the dosimeter was exposed to a radiation from a ^60^Co source, Fe^2+^ ions were gradually converted to Fe^3+^ ones. Analysis of UV-Vis spectra demonstrated that the intensity of the absorption peak of Fe^3+^ at 304 nm linearly increased in the range of the γ-radiation doses of 0–100 Gy. As compared to gelatin, PEO has a relatively simple molecular structure, which results in a single sharp peak at 3.464 ppm on the ^1^H NMR spectrum. Spin-echo NMR data demonstrate that R2 (1/T2) increased with the increase in the ionizing radiation dose. This effect was observed because of the conversion of Fe^2+^ to Fe^3+^ ions affecting the dipole interaction of the PEO protons.

Introduction of organic ligands is another effective method to hinder the diffusion of Fe^3+^ ions in Fricke gel dosimeters [54,55]. Irradiation-induced Fe^3+^ can chelate iminodiacetic acid groups in xylenol orange (XO) molecules, resulting in an obvious color change. Smith et al. introduced XO-modified PVA into gelatin to construct a novel Fricke gel dosimeter [55]. The optical dose response sensitivity of this dosimeter in the range of 0–32 Gy was 0.0031 Gy^−1^, its self-oxidation rate was 0.00023 h^−1^ and the diffusion rate was 0.132 mm^2^ h^−1^, respectively. The reported results demonstrate superior characteristics of the proposed dosimeter as compared to those of traditional Fricke gelatin ones. However, due to steric hindrance, Fe^3+^ ions cannot simultaneously complex with two iminodiacetic acid groups in XO. Moreover, XO prevents the coordination between Fe^3+^ ions and water molecules, thus weakening the resolution of dose measurements and the measurement accuracy at low radiation doses. To solve this issue, Lazzaroni et al. prepared an XO mimic with a single iminodiacetic acid group [56]. In such a system, a ligand forms a single stoichiometry Fe^3+^-ligand complex, which favors the reduction in the optical artifacts of gel dosimeters and thus, the improvement of the signal-to-noise ratio in the MRI analysis. In addition, Gallo et al. found that the pH significantly influenced the sensitivity as well as the linearity of the PVA Fricke gel dosimeter, whereas the gelation temperature did not [54]. To eliminate side-effects arising from temperature, Maeyama et al. prepared nanocomposite Fricke gel dosimeters with different concentrations of nanoclay, perchloric acid and ferrous ions in deaerated conditions [57]. As-prepared Fricke dosimeters can operate under both acidic and neutral conditions. Fricke dosimeters can be used as a tool in quality assurance in radiotherapy. For instance, a Fricke-XO gel dosimeter reported by Soliman et al. was calibrated in a phantom under exposure to 6 and 15 MV beams of the clinical linear accelerator. They found that the dose measurements with the dosimeter were comparable to that obtained by treatment planning systems and commercial ionization chambers [58].

**Figure 4:**
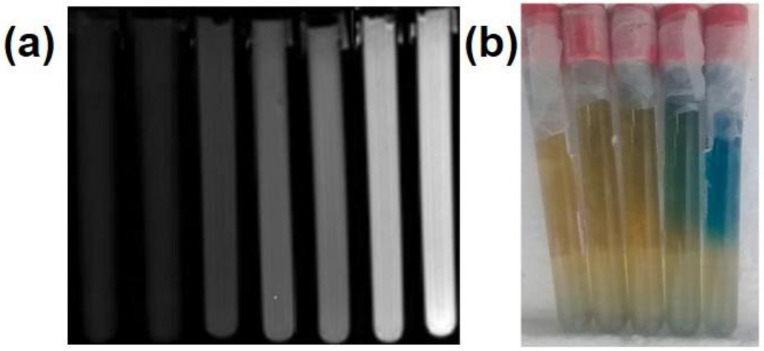
(**a**) MR and (**b**) digital images of dosimeters absorbed different doses. In (**a**) 0, 0.2, 1, 3, 4.5, 8 and 10 Gy and (**b**) 0.5, 1, 2, 5 and 10 Gy (from left to right), adapted from ref. [61] with permission from Elsevier.

Methyl thymol blue (MTB) is another common ligand in Fricke gel dosimeters [59,60,61]. As shown in Figure 4a, the MTB-Fricke dosimeter shows a gradual increase in turbidness in MR imaging with the increase in the radiation dose, and the irradiation of samples caused changes in the R2 value [61]. Due to the synergistic effect of MTB and benzoic acid (BA), the R1 and R2 sensitivity values of 0.058 ± 0.003 and 0.092 ± 0.004 s^−1^ Gy^−1^, respectively, of the MTB-BA-Fricke gelatin dosimeter in the radiation dose range of 0–10 Gy, were achieved. Figure 4b shows the photograph of the samples after irradiation to evaluate the diffusion of Fe^3+^ ions. The un-irradiated area is on the bottom side of the photograph while the top area of the vials is irradiated with different doses such that the authors can calculate the diffusion coefficient of the dosimeter. The diffusion coefficient of Fe^3+^ ions at the doses above 2 Gy was below 0.85 ± 0.02 mm^2^ h^−1^. Temperature, irradiation energy (6, 10, and 15 MV) and dose rate (0.65 to 5.25 Gy min^−1^) had a negligible influence on the dosimeter performance. Moreover, the UV-Vis absorption peak of Fe^3+^-MTB red-shifted to ~625 nm, and the light scattering decreased compared to that for Fe^3+^-XO. Both observed effects are beneficial for OCT imaging. The advantage of MTB-PVA-Fricke dosimeters is low autoxidation rate [56]. On the other hand, however, they have reduced absorbance sensitivity. The addition of chemical crosslinking agents such as glutaraldehyde (GTA) can improve the sensitivity, rigidity and thermal stability of such dosimeters [62]. Colnot et al. studied two commercial radiochromic gel dosimeters, TruView™ and ClearView™; the authors found both dosimeters presented a linear dose response up to 20 Gy and up to 80 Gy, respectively [63]. They both were independent of beam energy and dose rate. TruView™ is reusable and presented a excellent reproducible response, while the ClearView™ dosimeter presented a good spatial stability.

### 2.3. Radio-Chromic Gel Dosimeters

**Figure 5:**
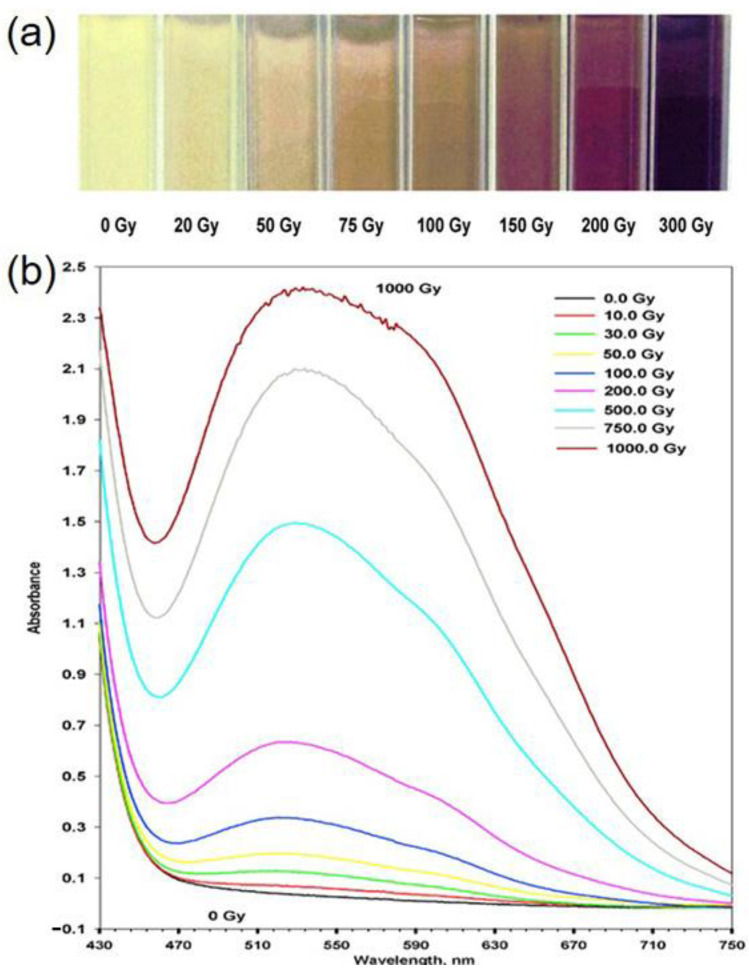
(**a**) Digital images and (**b**) UV-Vis spectra of NBT gel dosimeters, adapted from ref. [64] with permission from Elsevier.

The introduction of radio-chromic organic dyes such as methylene blue, azobenzene and nitro blue tetrazolium (NBT) is a common strategy for the fabrication of gel dosimeters. These organic dyes undergo a cleavage reaction after irradiation, causing the change in their color. The absorbed radiation dose can be measured by monitoring the ultraviolet spectra of the gel. Abdek-Fattach et al. introduced NBT into gelatin, and ethanol was added to improve dosimeter sensitivity [64]. As the irradiation dose increased from 10 to 1000 Gy, NBT gradually reduced to formazan and then to diformazan. The gel color changed at this from light yellow to lavender and finally to blue-purple (Figure 5). The maximum absorption peak of UV-Vis appeared at 527 nm. Such a dosimeter had a good tissue equivalence, temperature stability and low uncertainty.

Recently, Rabaeh et al. reported a novel radio-chromic PVA membrane embedded in methyl thymol blue. The characteristic absorption peak of MTB decreased with the increase in irradiation dose from 2.5 to 20 kGy [65]. Pilařova et al. reported a turnbull blue (TB) gel dosimeter, which consisted of a gel matrix, potassium ferricyanide and iron compounds dissolved in an acidic medium [66]. Irradiation of the gel in this dosimeter by γ-rays induced a reduction in Fe^3+^ ions into Fe^2+^ ones and the formation of K[FeII-FeIII(CN)_6_]. The absorption peak maximum of TB gel appeared at 690 nm and its intensity was proportional to the absorbed dose. This dosimeter could absorb the radiation at the doses in the range of 0–140 Gy.

### 2.4. Radio-Fluorogenic Gel Dosimeters

**Figure 6:**
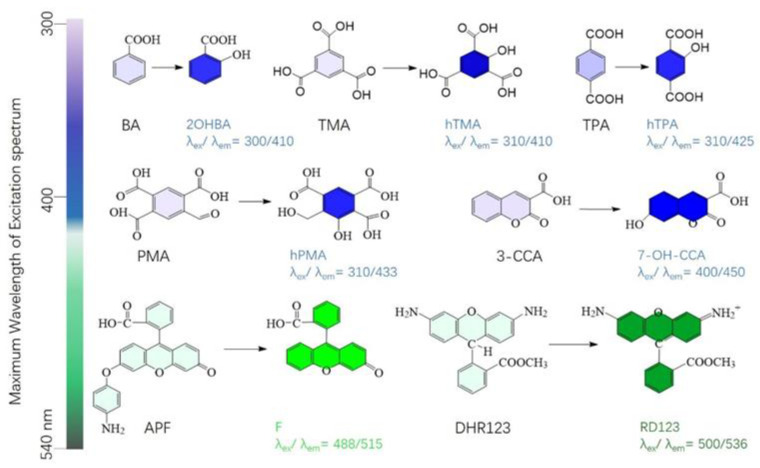
Representative radio-fluorogenic molecules.

Fluorescent gel dosimeters have attracted great attention due to their high sensitivity and good selectivity. A common way to fabricate such dosimeters is to add an ionizing radiation-responsive fluorescent probe to the gel matrix. For instance, Maeyama et al. reported on dosimeters constructed by incorporation of different fluorescent probes such as BA, trimesic acid (TMA), terephthalic acid (TPA), pyromellitic acid (PMA), coumarin-3-carboxylic acid (3-CCA), aminophenyl fluorescein (APF) and dihydrorhodamine 123 (DHR123) into nanoclays (Figure 6) [67,68]. Under ionizing radiation, water molecules undergo radiolysis and produce reactive oxygen species, which combine with these probes and form strongly fluorescent substances. It was found that the radio-fluorescent gel-based dosimeter has a good linearity in the dose range of 0–5 Gy. To enhance the sensitivity of fluorescent clay-based gel dosimeters, surfactants, which included polyethylene glycol octylphenyl ether (Triton X-100), sodium dodecyl sulfate, and cetyltrimethylammonium bromide (CTAB), were introduced to them. Furthermore, halides such as trichloroacetic acid, tribromoacetic acid and 2,2,2-trichloroethanol were also found to improve the dosimeter sensitivity [69]. It should be noted that pH significantly affects the peak excitation and the sensitivity of the radio-fluorogenic gel dosimeters [70].

**Figure 7:**
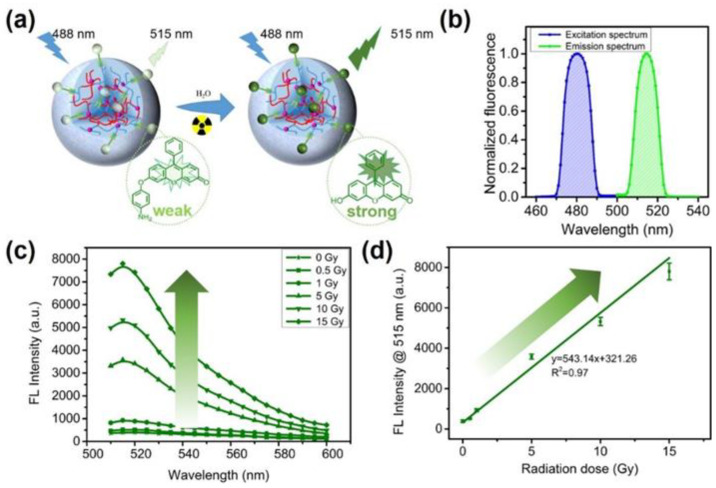
(**a**) Schematic illustration of the sensing mechanism and (**b**) the normalized FL spectra of PAAm-based nanogel dosimeters in response to X-rays. (**c**) The FL emission spectra and (**d**) FL intensity at 515 nm of the dosimeters with increasing X-ray doses. Adapted from ref. [72] with permission from American Chemical Society.

In recent years, our group has been working on fluorescent gel dosimeters. In one example, we prepared a PAAm-based fluorescence colorimetric nano-gel dosimeter combined with a radiation-sensitive fluorescent probe 3-CCA and a radiation-inert reference dye [5(6)-carboxytetramethylrhodamine, TAMRA] [71]. The irradiation of this dosimeter induced the reaction of the 3-CCA fluorescent probe with hydroxyl radicals formed by the radiolysis of water, yielding fluorescent 7-hydroxyl-coumarin-3-carboxylic acid (7-OH-CCA, λ_em_ = 450 nm). By contrast, the fluorescence of TAMRA was stable under X-ray irradiation. The fluorescence ratio of 7-OH-CCA to TAMRA increased linearly with the increase in the dose of X-rays from 0 to 20 Gy. The value of LOD was measured to be 0.1 Gy. Due to its nanoscale size and positive Zeta potential, the nanogel dosimeter is suitable to measure intracellular doses, thus providing a pathway for targeted therapy. In another report, fluorescent probe APF was connected to PAAm-based nanogels (Figure 7) [72]. After irradiation, APF attained a fluorescence property and emitted light at 515 nm. It was found that the nanogel dosimeter could detect X-rays at the doses of 0–15 Gy with the LOD value of 0.5 Gy. The fluorescence signal attenuation was up to 5% in the temperature range of 25–65 °C and only 9% after a 5-week period. Using the ocular sphere model, the lens and vitreous dose measurements were carried out under clinical conditions using the dosimeter considered. The obtained results were comparable with the ones of clinical treatment planning systems.

### 2.5. Gel Dosimeters with Embedded NPs

Owing to their unique physicochemical properties [73], metal nanoparticles have been widely used in the field of the measurement of ionizing radiation doses. Walker et al. used a mixture of HAuCl_4_ with cysteine-containing polypeptides and isopropyl alcohol. A total of 80–150 nm Au NPs were produced in this mixture under X-ray irradiation by the reduction in HAuCl_4_ [74]. The resultant NPs were chestnut colored and had the absorption peak maximum at 520 nm. The color of the dosimeter became darker and the absorption peak maximum linearly increased with the increase in X-ray dose in the range of 0 to 1072 Gy. Under the action of ionizing radiation, water molecules split to form hydroxyl, hydrogen radicals and hydrated electrons. Isopropanol reacted with the hydroxyl and hydrogen radicals and acetone reacts with hydrated electrons, resulting in the formation of 2-propyl radicals, which reduced Au(III) to Au(0). Under the action of the polypeptide template, Au NPs gradually nucleated and eventually formed.

**Figure 8:**
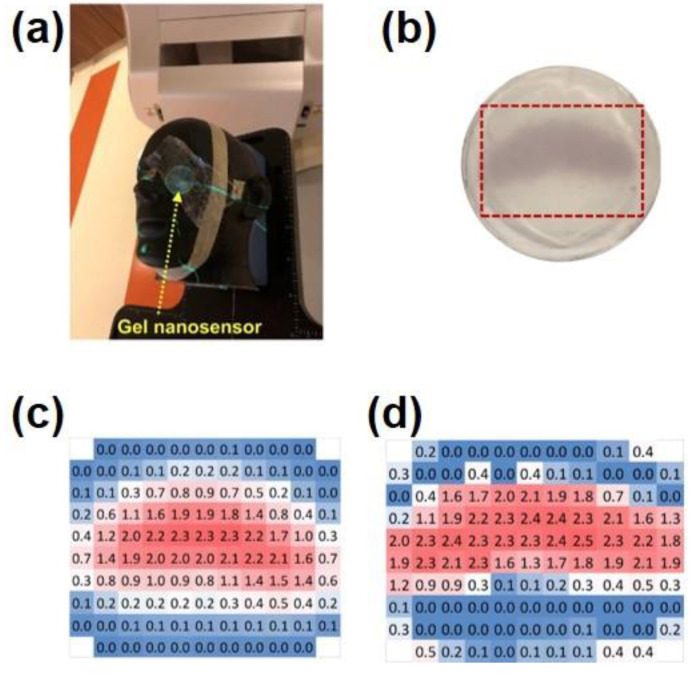
(**a**) A gel dosimeter mimics a conventional course of radiation therapy under the human left eye; (**b**) visual image of the dose pattern on the gel nanosensor formed after delivery of 2.3 Gy. (E) The core of the treated area with the expected crescent shape receives a radiation dose of 2.3 Gy (highlighted in red), and the areas receiving the lower dose are highlighted in different colors. (**c**) Expected topographical dose “heat map” profile of the radiation dose delivered to the gel placed in the phantom. (**d**) Radiation doses obtained from actual tests were broadly in line with expected estimates. Adapted from ref. [77] with permission from the American Chemical Society.

Inamdar et al. reported an Au NPs-embedded hydrogel dosimeter for dose measurements in hypersegmented proton radiotherapy [75]. In order to reduce the value of LOD, the authors introduced AA as an agent to reduce Au(III) to Au(I). Meanwhile, the cationic emulsifier, CTAB, was used as the template for the formation of Au NPs. Two hours after irradiation by a proton beam, Au(I) wrapped in agarose was further reduced and gradually formed Au NPs, which were accompanied by a color change to chestnut. The maximum absorption peak of the gel linearly increased in the dose range of 0–3 Gy. The dosimeter was finally placed in the radiotherapy equipment to test its clinical transformation capability. Being exposed to the radiation dose of 1.8 Gy, it showed a value of 1.6 Gy, which corresponded to the error of 11%. This error is comparable to the one of the clinical MOSFET dosimeters (Best Medical Canada, Ottawa, ON, Canada) of 5% [76]. As shown in Figure 8, the same group used gel dosimeters to perform two-dimensional dose mapping in an anthropomorphic head and neck model [77]. A total of 10 min after irradiation by the dose of 4 Gy, the gel turned purplish red. An hour later, the non-irradiated area also attained the same color, indicating the formation of Au NPs. This observation seriously affected the reliability of two-dimensional dosimetry. The authors suggested that the formation of additional Au NPs in non-irradiated areas took place mainly due to the seeding action of Au NPs and their reaction with Au(I). To solve this problem, Na_2_S was added about 10 min after the irradiation in order to convert Au(I) in the non-irradiated areas into Au_2_S. In this way, color diffusion was inhibited. The gel dosimeter was attached to the phantom and exposed to the radiation dose of 2.3 Gy. The measured dose results obtained by this dosimeter were close to the clinical treatment-planning system ones. Only the irradiated areas developed a maroon color, while the non-irradiated ones remained colorless.

In addition to Au NPs, Ag NPs can be also formed under ionizing radiation in the presence of AgNO_3_ and stabilizers (e.g., PVA, PVP) [78,79,80]. Soliman et al. mixed 200 mM AgNO_3_ with gelatin, fabricating Ag NP-based gel dosimeters [81]. Gelatin encapsulates and stabilizes Ag NPs. Under the γ-ray irradiation, Ag^+^ was reduced to Ag0 by hydrated electrons, and Ag0 gradually aggregated to form spherical NPs with the sizes ~65 nm. At this, the gel dosimeter turned yellow. A further increase in the radiation dose caused the change of the dosimeter color to yellowish-brown, and the characteristic absorption peak of the SPR of Ag NPs appeared at ~450 nm. The dosimeter has a dose detection range of 5–100 Gy with a minimum LOD value of 5 Gy. Furthermore, isopropanol [(CH_3_)_2_CHOH] is often used as a component of such dosimeters as a sacrificial agent for OH radicals to prevent the oxidation of Ag(0) by them [78]. It can react with H and OH radicals to produce [(CH_3_)_2_C∙OH], as well as having the ability to reduce Ag^+^.

## 3. Conclusions

In this review, recent advances of hydrogel sensors for measuring ionizing radiation doses are summarized. Various examples of hydrogel-based dosimeters, including polymer, Fricke, radio-chromic, radio-fluorogenic and NPs-embedded ones are reviewed. Hydrogel dosimeters are expected to have many features, including good sensitivity, accuracy and dose resolution. Many examples have demonstrated that their responses are independent of the beam type, energy and dose rate. These merits make gel dosimeters suitable in hospitals for dosimetry, although few commercial products are available.

Being traditional gel dosimeters, polymer and Fricke dosimeters should be prepared with complex gel formulations in an oxygen-free environment, with the corresponding difficulty in reproducibility. Moreover, these dosimeters need to be stored at temperatures as low as 4 °C, and usually require the use of expensive MRI readout systems. The toxicity of monomers imposes some limitations in the production process and routine clinical applications (i.e., use and disposal). Therefore, radio-chromic dosimeters, as well as radio-fluorescence and NPs-embedded dosimeters, have been studied. The addition of fluorescent probes to the hydrogel matrix opens a new and simple way to measure ionizing radiation doses by analyzing fluorescence spectra. In these cases, the pH and temperature of the gel-dosimeters may significantly affect their properties. The use of nanomaterials with high atomic numbers of composing elements improves their sensitivity and reduces LOD values. Unfortunately, the design and fabrication of such dosimeters require complex preparation steps with the accuracy and reproducibility still to be improved. To achieve the goal of a wide use of hydrogel dosimeters for clinical measurements of radiation doses, more experts from various fields, including materials science, chemistry, cancer therapy, radiobiology and nuclear science, should be involved. In brief, chemists need to explore easy and robust preparation methodologies and oxygen-/light-/heat-tolerant monomers/polymers. Doctors and radiotherapy physicists can help to test the capability of gel dosimeters for clinical quality assurance and quality control. Exports from materials science and nuclear science should aim to develop novel gel systems and devices for dosimetry.

## Figures and Tables

**Table 1:** Characteristics of representative polymer dosimeters.

Dosimeter	Main Composition	Beam	Linear Range (Gy)	LOD (Gy)	Measurement
BANANA	AAm, BIS, agarose [22]	X-ray	0–20	~4	MRI
BANG	AAm, BIS, gelatin [23]	X-ray	0–8	1	MRI
BANG-3	AAc, BIS, gelatin [24]	γ-ray	0.5–3.5	0.5	MRI
		neutron	0–2.5	<0.5	
nPAG	AAm, BIS, gelatin [25]	X-ray	0–5	0.2	SPR
U-NIPAM	NIPAm, BIS, agarose [26]	X-ray	0–7	1	MRI
PAMPSGAT	AMPS, BIS, gelatin [27]	γ-ray	10–40	10	MRI
MAGIC	MAA, AA, gelatin [28]	X-ray	0–16	4	MRI
MAGT-A	MAA, gelatin, agar [29]	X-ray	0–10	2	MRI
MAGAT	MAA, gelatin [30]	X-ray	1–10	1	UV-Vis
DEMBIG	DEMA, BIS, gelatin	X-ray	0–20	5	MRI [31]
DEMBIG	DEMA, BIS, gelatin	X-ray	0–30	1	Laser optic tomography [32]
DEMBIG	DEMA, BIS, gelatin	X-ray	1–25	1	CT [33]
NIBMAGAT	NIBMA, BIS, gelatin [34]	X-ray	0–10	2	MRI
NHMA	NHMA, BIS, gelatin [35]	X-ray	0–6	1	MRI

Abbreviation. AAm: acrylamide, BIS: N, N′-methylene bisacrylamide, NIPAm: N-isopropylacrylamide, AMPS: 2-acrylamido-2-methylpropane sulfonic acid, MAA: methacrylic acid, AAc: acrylic acid, DEMA: 2-(Dimethylamino) ethyl acrylate and AA: ascorbic acid. NIBMA: N-(isobutoxymethyl)acrylamide.

## Data Availability

Not applicable.
